# Management of foreign bodies ingestion in children

**DOI:** 10.1007/s12519-022-00585-7

**Published:** 2022-08-05

**Authors:** Qing-Jiang Chen, Lin-Yan Wang, Yi Chen, Jia-Jin Xue, Yue-Bin Zhang, Li-Feng Zhang, Yun-Zhong Qian, Qi-Xing Xiong, Zhi-Gang Gao

**Affiliations:** Department of Pediatric General Surgery, National Center for Clinical Medicine of Children’s Health and Disease, Children’s Hospital, Zhejiang University School of Medicine, National Children’s Regional Medical Center, 3333 Binsheng Road, Binjiang District, Hangzhou, 310052 China

Foreign bodies (FBs) ingestion in children is one of the common presentations to the emergency department. The majority of FBs ingestion occurs in children younger than 3 years, with males showing a slight predominance [1]. Although most FBs pass through the gastrointestinal tract spontaneously, some FBs need urgent medical intervention and even surgical management owing to the complications of obstruction or perforation and a potential risk to children’s health [2]. Therefore, timely diagnosis and rational treatment are crucial for reducing FBs complications. However, optimal indications and/or timing of surgery remain controversial. In this study, we discussed the characteristics of FBs ingestion in children and summarized our experience in its management.

We reviewed the patients diagnosed with FBs ingestion and admitted to the Department of General Surgery, Children's Hospital of Zhejiang University School of Medicine between January 2010 and December 2020, focusing on clinical, radiologic, and prognostic findings. The patients with FBs ingestion that needed surgical intervention were included in this study, whereas patients with conservative treatment or endoscopically managed were excluded. Statistical analysis was completed using the Statistical Program for Social Sciences (SPSS, version 19.0, Chicago, IL) to perform analysis of variance (ANOVA) or the chi-square tests; *P* < 0.05 was considered to be statistically significant. The project was approved by the Ethics Committee of the Hospital(2022-IRB-095).

This study included 119 patients with ages ranging from 8 months to 13.83 years (median 3.67 years). Forty-nine cases had a definite history of FBs ingestion, with a residence time varying from 10 h to 5 years. The rest of the 70 patients were identified under imaging examination or surgical exploration. The FBs were located in various parts of the digestive tract, with 25 cases of magnetic FBs being multiple positioned. Ninety patients were symptomatic and presented with vomiting (80 cases), stomachache (72 cases), abdominal tenderness (47 cases), abdominal distention (26 cases), fever (20 cases), palpable mass (14 cases), muscle guarding (7 cases), perianal pain and redness (1 case). Eighty-two patients had FBs-related complications, including gastrointestinal perforation (47 cases), intestinal obstruction (34 cases), and fistula-in-ano (1 case) (Table 1). All cases of intestinal obstruction and 8 cases of gastrointestinal perforation were diagnosed preoperatively by imaging or gastroscopy; the remaining 40 of FBs-related complication were verified during surgical exploration.

**Table 1:** Detailed features of gastrointestinal foreign bodies (FBs)

Type of FBs	Cases	Location	Surgical procedures	FBs-related complications	Associated anomalies
Magnetic beads	55	Stomach, duodenum, intestine, colon	Enterotomy, enterectomy, perforation repair	Bowel perforation (44)	–
Phytobezoar	26	Intestine	Enterotomy or push into colon	Intestinal obstruction (26)	MD (1)
Trichobezoar	17	Stomach (14), intestine (3)	Gastrotomy or enterotomy	Intestinal obstruction (3)	–
Water-absorbing beads	4	Distal ileum	Enterotomy	Intestinal obstruction (4)	–
Screw	2	Appendix (1), MD (1)	Appendectomy or diverticulectomy	–	MD (1)
Coins	2	Duodenum (1), jejunum (1)	Duodenotomy, jejunal web resection (1)	–	Jejunal web
Chicken bone	2	Rectum	Transanal, thread-drawing (1)	Perianal abscess and fistula (1)	–
Paperclip	2	Duodenum	Push back to stomach	–	–
Date stone	2	Distal ileum	Perforation repair, enterectomy	Ileum perforation (2)	–
Hairpin	1	Ileum	Enterotomy	–	–
Nail	1	Ileum	Enterotomy	–	–
Dental needle	1	Ileum	Enterotomy	–	–
Safety pin	1	Ileum	Push into rectum	–	–
Metal ring	1	Rectum	Transanal remove and anoplasty	–	Anus stenosis
Thumbtack	1	Ileum	Enterectomy	Ileum perforation	–
Litchi	1	Ileum	Enterotomy, enterectomy	Intestinal obstruction	–

*MD* Meckel’s diverticulum, *–* none

In the group of 47 cases with gastrointestinal perforation, 25 of abdominal pain, 29 of vomiting, 12 of fever and 9 of abdominal distention were presented. No significant differences in the incidence of symptoms (including abdominal pain, vomiting and abdominal distention) were observed as compared with non-perforated group (*X*^*2*^ = 1.738, 1.076, 0.332, and *P* = 0.250, 0.323, 0.653, respectively), except fever (*P* = 0.048). No significant differences in white blood cell (WBC) counts (11.79 ± 3.69 vs 11.30 ± 3.91, *P* = 0.654)and percentage of neutrophils (61.68 ± 17.05% vs 69.06 ± 16.62%, *P* = 0.114) were detected in the perforation group as well 34.00% (16/47) patients with gastrointestinal perforation had an elevation of serum C-reactive protein (CRP) level (> 8 mg/L), more obvious than the non-perforated patients (30.60%, 22/72), but no difference was observed (*X*^*2*^ = 0.159, *P* = 0.693).

In children, significantly increased magnetic FBs ingestion has been reported in recent years [3, 4]. When multiple magnetic FBs are ingested, these magnets have the potential to attract each other across the bowel and result in tissue necrosis, formation of fistula, and obstruction or intestinal perforation [4]. In our study, among the 55 cases of magnetic FBs, 44 had the complication of intestinal perforation, and 10 had an intestinal obstruction. However, only 4 cases of magnetic FBs with pneumoperitoneum were observed under abdominal radiography or computed tomography (Fig. 1). Huang et al. reported that no sub-diaphragmatic free air was identified in 10 cases of magnetic FBs with intestinal perforation [5]. Most patients were detected with intestinal fistula during operative exploration, without obvious signs of peritonitis and significantly increased WBC or CRP level.

**Fig. 1:**
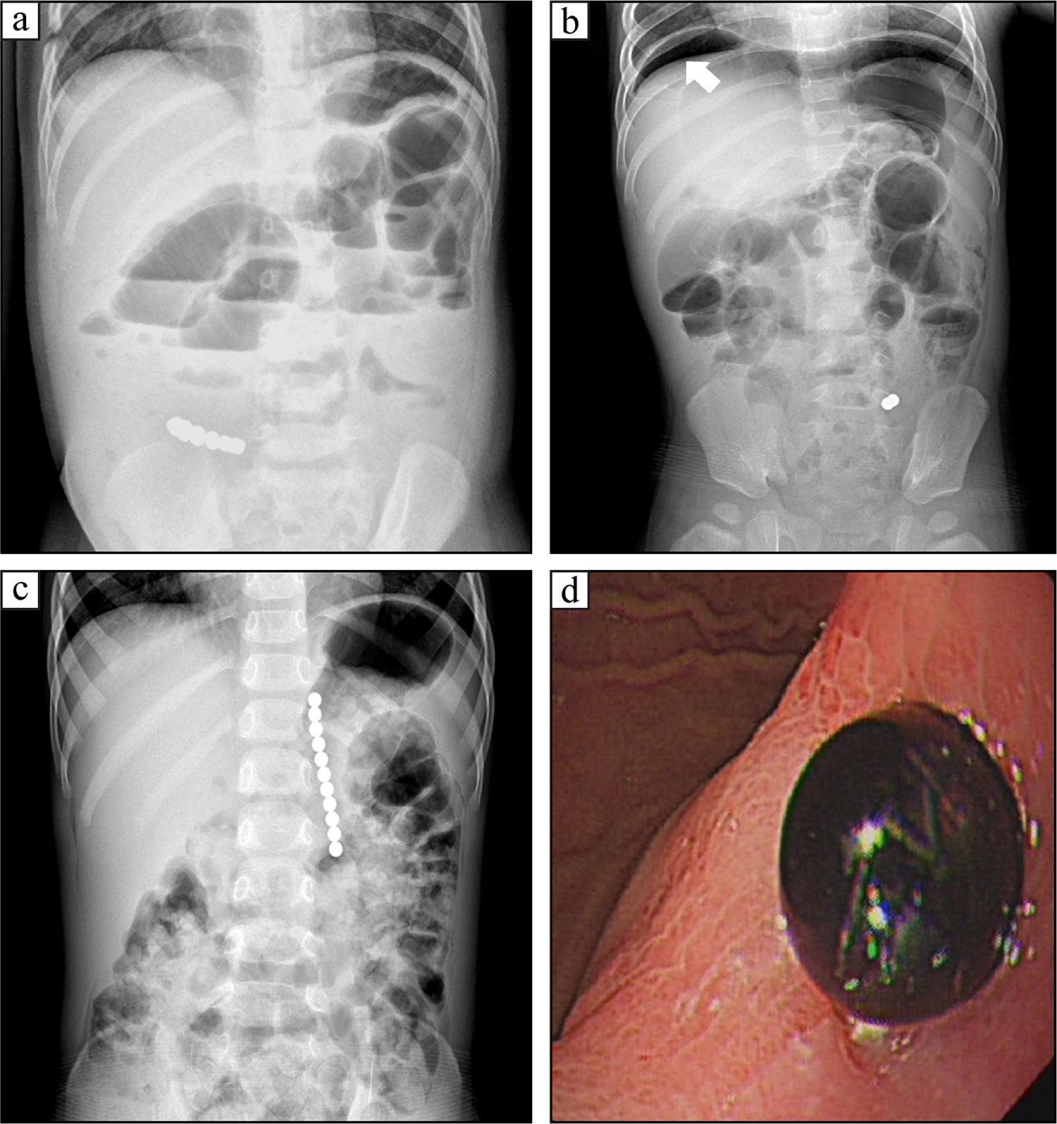
Magnetic foreign bodies (FBs) ingestion. **a**: Multiple magnetic FBs complicated with intestinal obstruction; **b**: Magnetic FBs with intestinal perforation, with sub-diaphragmatic free air (white arrow head); **c**: Multiple magnetic FBs in the left upper quadrant; **d**: Magnetic beads trapped into the gastric wall and with under gastroscopy in the patient perforation shown in Fig. 1c

Bezoars are the accumulation of indigestible materials, such as hair, vegetable, and fruit, in the gastrointestinal system. Immature persimmons, prunes, and Chinese dates are rich in tannic acid, gum, and pectin, which form an adhesive-like substance when they encounter gastric acids. Further, they hold other plant fibers together, resulting in the formation of phytobezoars and subsequent ileus [6]. In our series, 26 cases of phytobezoars were identified during abdominal exploration due to intestinal obstruction, and only three reported the possibility of FBs by preoperative imaging. Trichobezoars are commonly encountered in adolescent girls with psychiatric illnesses who desire to pluck and ingest hair. In these patients a large quantity of hair remained in the stomach folds, intermixed with food and other fibers to form a gastric-shaped bezoar, and may extend to the small bowel. These bezoars are commonly presented as an abdominal mass or as features of bowel obstruction, often misdiagnosed as an abdominal tumor. Therefore, surgery is usually indicated, and laparotomy is still preferred [7]. Seventeen cases of trichobezoars were diagnosed in our patients. All were girls, with a mean age of 9.01 ± 1.63 years. Fourteen and three cases of trichobezoars were located in the stomach and the small intestine, respectively, with symptoms of abdominal pain in 16 cases, vomiting in 11 cases, and palpable abdominal mass in 14 cases. No significant psychiatric ailments were identified in our cases. All patients were preoperatively diagnosed with stomach trichobezoars or small bowel FBs by endoscopy, ultrasonography, and/or computed tomography (CT) (Fig. 2). Though endoscopic removal is the most attractive choice, successful endoscopic removals are remarkably scarce [7].

**Fig. 2:**
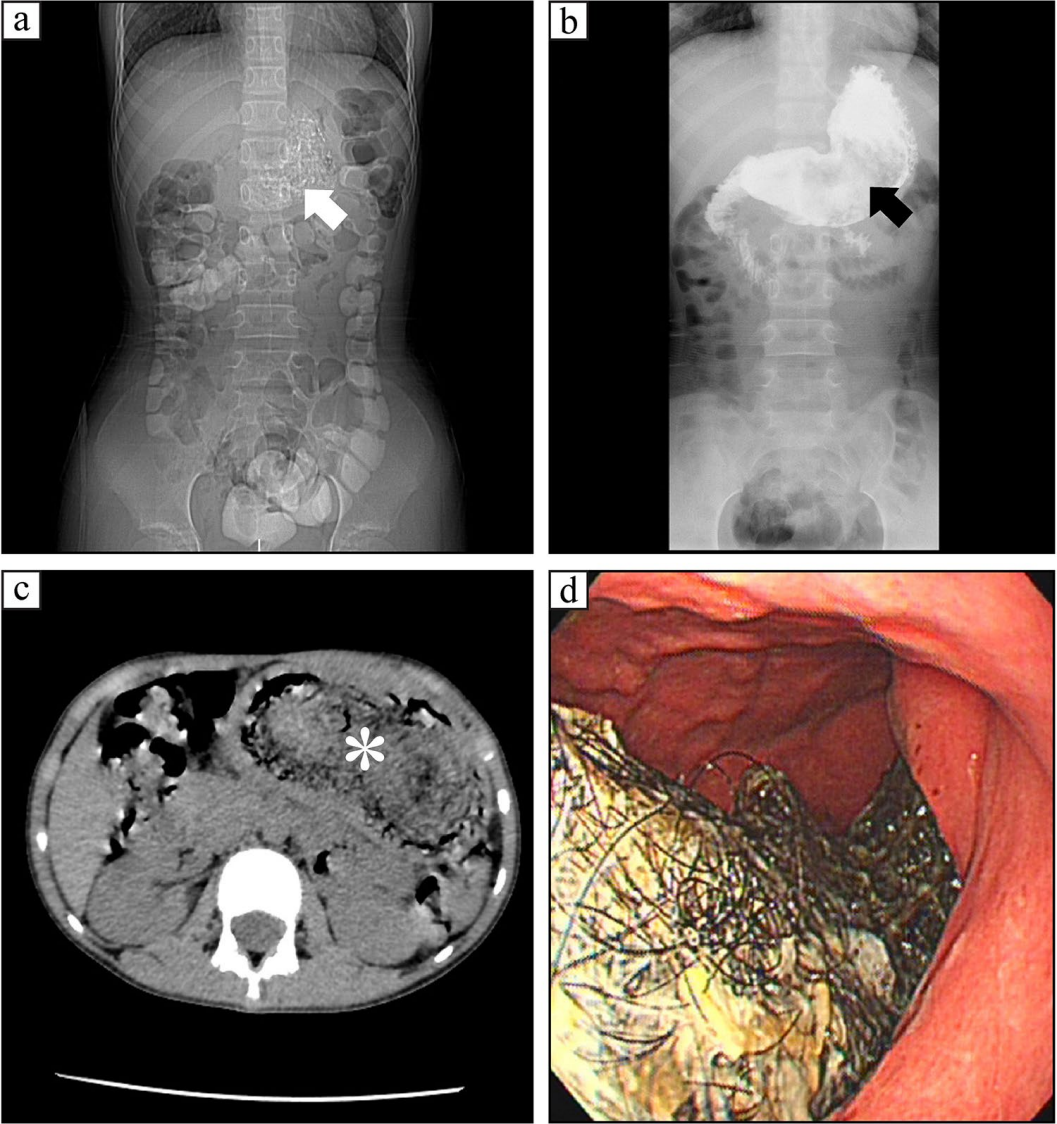
Gastric trichobezoars. **a**: Abdominal plain film showing a heterogeneous density mass in the left upper quadrant (white arrow head); **b**: GI demonstrating a gastric filling defect (black arrow head); **c**: CT scanning displaying a miscellany density mass in the stomach (asterisk); **d**: Gastroscopy revealing a hair mass in the stomach and duodenum. *GI* Upper gastrointestinal study, *CT* computed tomography

Most FBs ingestions are asymptomatic, or sometimes the clinical symptoms are nonspecific, which may delay diagnosis and increase the risk of complications [8]. Imaging examination plays an important role in the assessment of ingested FBs. It can determine the type of object ingested, its location in the alimentary tract, and the presence of any associated complications. The plain radiograph is still the most commonly used radiologic procedure to recognize the ingested FBs in these children [9]. In this group, appropriate diagnostic workups were selected according to the pattern of clinical presentation in 118 patients. The plain abdominal film was carried out in 102 cases, which confirmed alimentary tract FBs in 60, intestinal obstruction in 45, and pneumoperitoneum in 5. CT scanning was performed in 24 cases and reported FBs in 18, intestinal obstruction in 6, pneumoperitoneum, and abdominal mass in 1 (Fig. 3). Out of eighteen patients who underwent gastroscopy, 12 of trichobezoar and 6 of magnetic beads with gastric perforation in 2 were verified. Only eight cases of gastrointestinal perforation were identified preoperatively. Four cases of water-absorbing bead ingestion presented with intestinal obstruction. Spherical anechoic mass and proximal intestinal dilatation were observed under ultrasound, mistaken for alimentary tract duplication in two cases (Fig. 3).

**Fig. 3:**
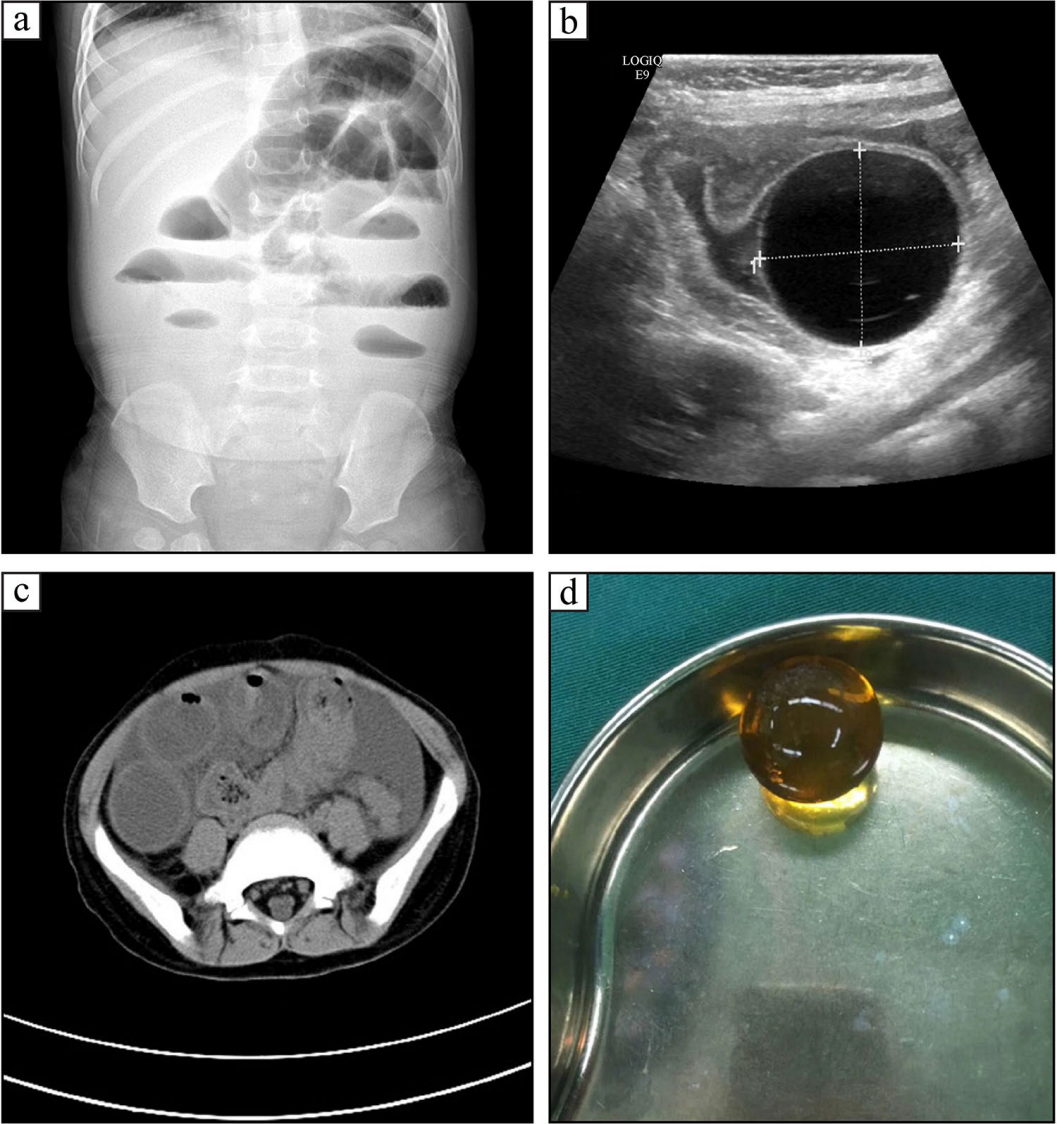
Water-absorbing bead ingestion. **a**: Abdominal radiographs indicating complete intestinal obstruction; **b**: Ultrasonography revealing a well-marginated hypoechoic mass in the intestinal cavity, with proximal intestinal dilatation; **c**: CT scanning showing intestinal obstruction without FBs detected; **d**: The water-absorbing bead was removed by surgery. *CT* computed tomography

Management of FBs ingestions varies based on the type and location of the FBs, the time since ingestion, as well as the patient’s age and size. Normally, 80–90% of FBs pass spontaneously through the GI tract, while 10–20% require endoscopic removal, and less than 1% need surgical intervention. However, some items of FBs, such as magnets and button batteries, carry significantly increased complication and thus increases the risk of surgery. Therefore, it is critical to determine the indication and timing of the operation before serious complications occur. Unfortunately, there are no universally implemented guidelines to deal with this concern in children. We formulated a simple algorithm that could be implemented by physicians to whom these patients present (Fig. 4). Prompt surgery is required for patients with FBs-related complications, such as bleeding, obstruction, or perforation. In this group surgical results demonstrated 47 cases with perforation, 34 with ileum, 31 with FBs impaction, and 1 with anal fistula. The remaining 11 cases with multiple magnets and symptoms of abdominal pain and/or vomiting were not associated with FBs complications due to timely operation. Our experience and the results reported in published data concluded that aggressive surgical treatment should be performed under demanding circumstances as followings [10, 11]: (1) FBs-related complications, such as massive hemorrhage, gastrointestinal perforation, and intestinal obstruction, are the absolute indications for operation; (2) Large intra-gastric FBs, such as trichobezoar, cannot be removed easily by endoscopy; (3) For objects that stay at the same location for more than one week or sharp-pointed objects that fail to progress for 3 consecutive days, surgical intervention should be considered; (4) Multiple magnetic FBs or magnetic FBs ingested with metallic FBs simultaneously are at increased risk of complications such as fistulation, obstruction, and perforation; thus, aggressive management, including surgical removal, is mandated, and (5) Symptoms of fever, vomiting, abdominal pain and distention, or bloody stool developed during conservative treatment, are important indications of intestinal injury, and immediate surgical evaluation is suggested.

**Fig. 4:**
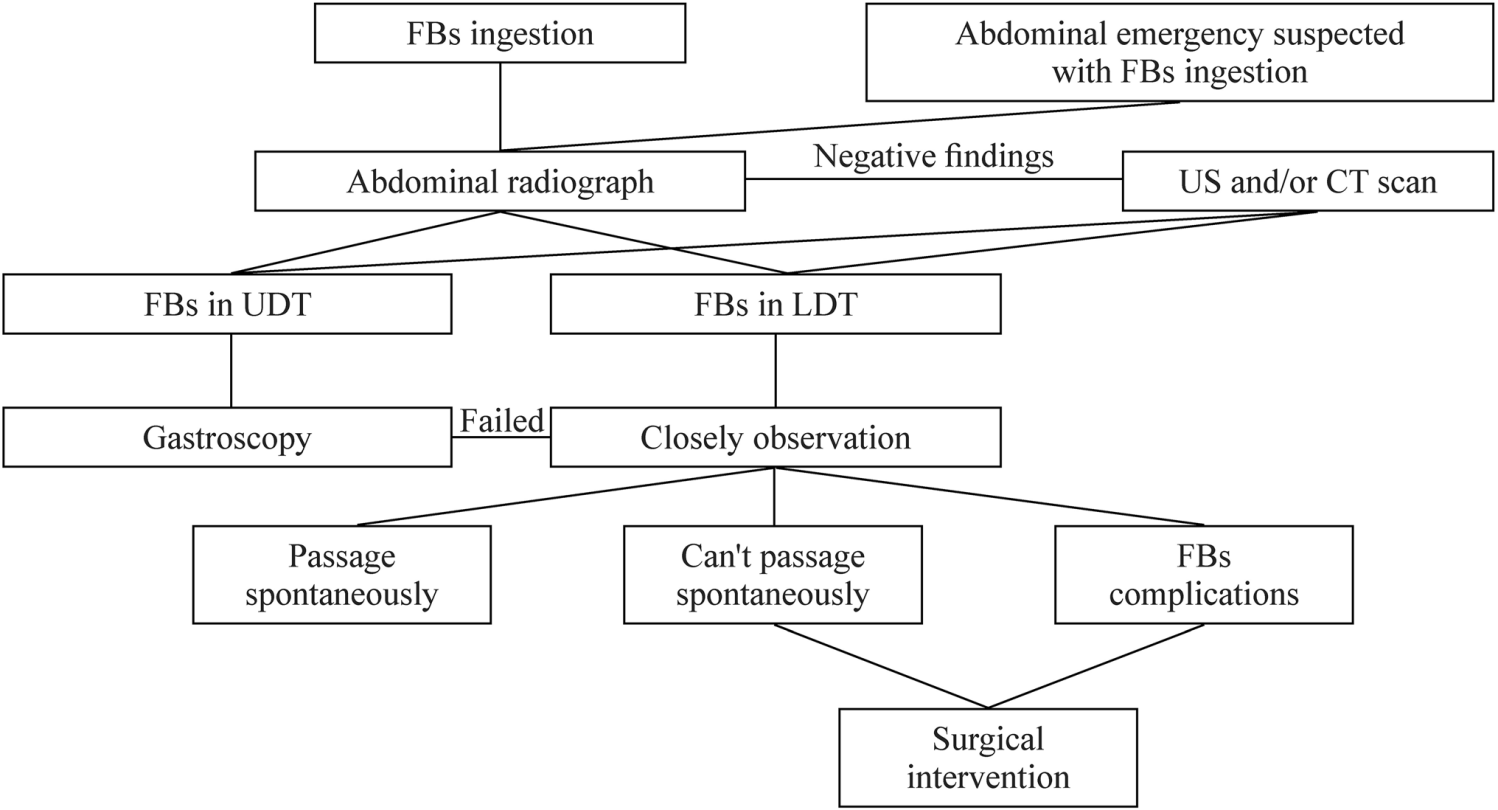
Suggested algorithm for children with FBs ingestion. *FBs* foreign bodies, *US* ultrasonography, *CT* computed tomography, *UDT* upper digestive tract, *LDT* lower digestive tract

Surgical procedures were selected according to the type and location of the FBs. Transanal approach was applied to 3 cases of rectal FBs. One hundred cases of laparotomy and 16 of laparoscopy were performed. The FBs were removed by gastroenterotomy in 108 cases, pushed into the colon in 5, pushed back into stomach and removed by gastroscopy in 2, removed by appendectomy or diverticulectomy in 1, respectively. Among the 47 cases of complicated perforation, 32 of multiple perforations or fistulae were identified; forty-two cases were treated with fistulectomy or perforation repair and 5 were managed with intestinal resection and anastomosis. Associated anomalies were corrected simultaneously, including 2 of diverticulectomy, 1 of jejunal web resection and 1 of anoplasty.

Though most FBs pass the gastrointestinal tract smoothly or can be removed by endoscopy easily, it still remains an important risk factor for children’s health. In our report, one patient with severe abdominal distention and gastric regurgitation died of respiratory failure and septic shock on the third postoperative day. Nine cases of postoperative complications were observed, in which 1 case of anastomotic leakage was reoperated, 4 of wound infection and 4 of adhesive ileus were managed conservatively. FBs ingestion is a significant public health problem in children. Public education and advocacy efforts should be strengthened to provoke greater awareness within the medical and parental communities to reduce the incidence of FBs ingestion.

There are several limitations to this study. First, it is a retrospective study. There are differences in the stage and severity of the disease at patients’ admission, which make it difficult in mapping the evolution of the disease; second, this study only included patients with FBs ingestion managed by surgery, while patients with conservative treatment or endoscopically managed were excluded; thus, this study cannot fully reflect the routine of diagnosis and treatment of FBs ingestion in children; third, the initial treatment plan of laparoscopy or laparotomy, push FBs into colon or enterotomy was partially based on the preference of the treating pediatric surgeon.

In conclusion, FBs ingestions are common abdominal emergency in children, and still pose a great threat to children's health. It is crucial to formulate a reasonable diagnostic and therapeutic strategy, master the surgical indications and opportunity strictly to improve the prognosis of the disease.

## Declarations

### Data availability

The data used to support the findings of this study are available from the corresponding author upon request.
